# Modulation of Th1/Th2 Immune Responses by Killed *Propionibacterium acnes* and Its Soluble Polysaccharide Fraction in a Type I Hypersensitivity Murine Model: Induction of Different Activation Status of Antigen-Presenting Cells

**DOI:** 10.1155/2015/132083

**Published:** 2015-04-20

**Authors:** Carla Cristina Squaiella-Baptistão, Daniela Teixeira, Juliana Sekeres Mussalem, Mayari Eika Ishimura, Ieda Maria Longo-Maugéri

**Affiliations:** ^1^Laboratório de Imunoquímica, Instituto Butantan, Avenida Vital Brazil 1500, Prédio Novo, 2° Andar, Butantã, 05503-900 São Paulo, SP, Brazil; ^2^Disciplina de Imunologia, Departamento de Microbiologia, Imunologia e Parasitologia, Universidade Federal de São Paulo, Rua Botucatu 862, Edifício de Ciências Biomédicas, 4° Andar, Vila Clementino, 04023-062 São Paulo, SP, Brazil

## Abstract

*Propionibacterium acnes* (*P. acnes*) is a gram-positive anaerobic bacillus present in normal human skin microbiota, which exerts important immunomodulatory effects, when used as heat- or phenol-killed suspensions. We previously demonstrated that heat-killed *P. acnes* or its soluble polysaccharide (PS), extracted from the bacterium cell wall, suppressed or potentiated the Th2 response to ovalbumin (OVA) in an immediate hypersensitivity model, depending on the treatment protocol. Herein, we investigated the mechanisms responsible for these effects, using the same model and focusing on the activation status of antigen-presenting cells (APCs). We verified that higher numbers of APCs expressing costimulatory molecules and higher expression levels of these molecules are probably related to potentiation of the Th2 response to OVA induced by *P. acnes* or PS, while higher expression of toll-like receptors (TLRs) seems to be related to Th2 suppression. *In vitro* cytokines production in cocultures of dendritic cells and T lymphocytes indicated that *P. acnes* and PS seem to perform their effects by acting directly on APCs. Our data suggest that *P. acnes* and PS directly act on APCs, modulating the expression of costimulatory molecules and TLRs, and these differently activated APCs drive distinct T helper patterns to OVA in our model.

## 1. Introduction

*Propionibacterium acnes* (*P. acnes*) is a gram-positive anaerobic bacillus present in normal human skin microbiota [1] but also implicated in inflammatory diseases, such as acne vulgaris [2] and sarcoidosis [3]. An important property of this bacterium is its immunomodulatory effect, which has been extensively studied in human and animal models, using heat- or phenol-killed* P. acnes* suspensions. Its main biological activities include sensitization to the toxic activity of lipopolysaccharide (LPS) [4], activation of macrophages [5], induction of tumoricidal activity [6], increase of the resistance to pathogens [7, 8], and adjuvant effect to antibody response [9]. These effects are mediated by proinflammatory cytokines, enhanced by* P. acnes* treatment, such as IFN-*γ*, TNF-*α*, IL-1*β*, IL-6, IL-12, and IL-18 [10–14]. The induction of these cytokines by* P. acnes* is dependent on TLR2, TLR9, and MyD88 [15–17]. The bacterium also enhances the expression of TLR2 and TLR4 by keratinocytes [18] and TLR4 and MD-2 by hepatocytes [19], what can explain its ability to potentiate the endotoxic shock to LPS. Recently, it was also demonstrated that* P. acnes* can activate the inflammasome of human peripheral neutrophils, as shown by caspase-1 increased expression [20].

Due to the cytokines pattern induced by* P. acnes*, it has been used as a Th1-inducer antigen [21–23]. However, in a previous study from our group, we demonstrated that heat-killed* P. acnes* could not only induce a typical Th1 response, but also enhance the Th2 pattern elicited by another antigen [24], using a murine model of type I hypersensitivity reaction previously described [25].

A soluble polysaccharide component purified from the bacterium cell wall also induced the same responses in such model [26]. This polysaccharide (PS) was purified and characterized by our group [26], and it had already been studied in other models, inducing similar effects to the whole bacterium, such as adjuvant effect to antibody response on a* Trypanosoma cruzi* DNA vaccine [27], increase of the number and tumoricidal activity of peritoneal macrophages [28], and enhancement of dendritic cells* in vivo* and* in vitro* [29]. Recently, PS was also shown to modulate the Th2 response observed in a murine model of focal segmental glomerulosclerosis, inducing a Th1 polarization and kidney preservation [30]. All these results suggest that PS can be a major* P. acnes* component responsible for its effects, including the polarization of T cell responses.

In the present data, we investigated the mechanisms by which* P. acnes* and PS polarize the immune responses in the type I hypersensitivity model cited above. In such model, described by Facincone et al. [25], F1 BALB/c x A/J mice are subcutaneously sensitized with heat-coagulated hen's egg white (HEW) and 14 days later challenged with heat-aggregated OVA in the footpad. A typical late phase reaction (LPR) of immediate hypersensitivity, with intense eosinophilia, is induced [25]. In our previous works using this model, we established two* P. acnes*- or PS-treatment protocols [24, 26]. In Protocol 1 (Th2 potentiation), mice were subcutaneously injected with* P. acnes* or PS once a week, during 3 weeks, and HEW was implanted concomitantly to the last* P. acnes* or PS injection. In Protocol 2 (Th2 suppression), HEW was implanted 1 week after the third dose of* P. acnes* or PS. For each protocol, the respective control group was treated with sterile saline, at the same conditions. Two weeks after HEW implantation, animals were challenged with heat-aggregated OVA in the footpad, as described above. Footpad swelling and histological analysis were performed, determining the number of eosinophils and other inflammatory cells infiltrating the footpad lesions. Intracellular and sera cytokines levels were also analyzed. Protocol 1 increased all the parameters evaluated, indicating Th2 potentiation, while Protocol 2 decreased the response, indicating Th2 suppression [24, 26]. These data clearly demonstrated that differences between the two protocols occur at the time of HEW implantation, indicating different environment in the moment of antigen capture by antigen-presenting cells (APCs).

Therefore, herein we focused our interest on APCs, which are responsible for T cell direction. We observed that the activation status of spleen B lymphocytes, macrophages, and dendritic cells was different depending on the treatment protocol with* P. acnes* or PS in the hypersensitivity model. Therefore, the Th1/Th2 polarization by the bacterium or its compound is related to the activation status of APCs, with different patterns of costimulatory molecules and toll-like receptors expression, leading to the production of different types of cytokines.

## 2. Material and Methods

### 2.1. Animals

Male or female 6-week-old F1 BALB/c x A/J mice were used in all experiments. Animals were housed in standard cages and kept on a 12-hour light/dark cycle, at controlled temperature, with water and food* ad libitum*. Experimental procedures were approved by the special University Ethics Committee for animal care and experimentation (CEP-1211/2004).

### 2.2. Antigens

*Heat-Killed P. acnes Suspension*.* P. acnes* (gently provided by Instituto Adolfo Lutz, SP, Brazil) was cultured in anaerobic medium (Hemobac, Probac, SP, Brazil) for 3 days, at 37°C. After this period, bacteria were washed three times at 2,000 g, for 30 minutes. The pellet was resuspended in 0.9% saline and subjected to continuous water vapor for 20 minutes, at 120°C. The protein concentration of the suspension was determined by the Bradford method [31].

*P. acnes Soluble Polysaccharide (PS) Extraction*. The polysaccharide extraction and purification were performed as previously described by us [26], based on Palmer and Gerlough protocol [32]. Briefly, 30 mL of heat-killed* P. acnes* (800 *μ*g of protein/mL) was mixed with 30 mL of 90% phenol and 30 mL of distilled water and incubated for 10 minutes in 70°C water bath. After centrifugation at 2,000 g and 4°C, the water phase and the polysaccharide-enriched ring were collected. This step was repeated three times. Three volumes of ethanol were added to each volume of the mixture. After overnight incubation at 4°C, the precipitate was obtained by centrifugation and designated as the soluble polysaccharide (PS). The carbohydrate concentration was determined by the Dubois method [33].

*Heat-Coagulated Hen's Egg White (HEW) for Mice Sensitization*. Heat-coagulated HEW was prepared as previously described [25]. The solid egg white was obtained after 30 minutes in boiling water; then, it was isolated, washed in distilled water, and dehydrated in 100% ethanol overnight. Fragments of approximately 40 mg were obtained and rehydrated in sterile PBS for 2 hours at room temperature, before implantation.

### 2.3. Type I Hypersensitivity Murine Model

The immediate hypersensitivity model used in our previous studies was developed by Facincone et al. [25]. Accordingly, when F1 BALB/c x A/J mice are subcutaneously implanted with heat-coagulated HEW (40 mg) and challenged, after 14 days, with 50 *μ*L of heat-aggregated OVA (20 mg/mL) in the footpad, they develop a late phase reaction of the type I hypersensitivity reaction, characterized by footpad swelling and eosinophil infiltration [25]. In the present work, mice were submitted only to the sensitization phase (HEW implantation) of type I hypersensitivity model.

### 2.4. *P. acnes*- or PS-Treatment Protocols

**Figure 1:**
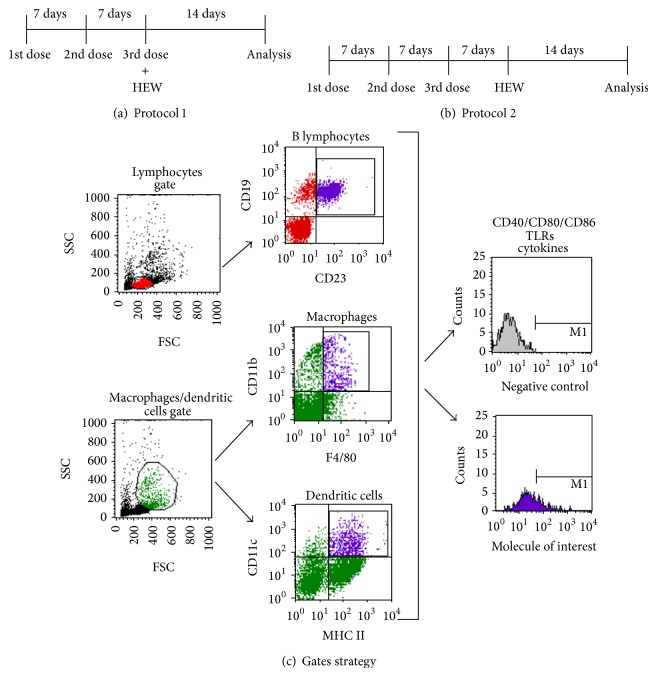
Schematic representation of* P. acnes*- and PS-treatment protocols and gates strategy. F1 BALB/c x A/J mice were subcutaneously injected with* P. acnes* or PS once a week, during 3 weeks. Heat-coagulated hen's egg white (HEW) was implanted concomitantly to the last* P. acnes* or PS dose in Protocol 1 (a) or 1 week after the third dose in Protocol 2 (b). For each protocol, the respective control group was treated with 350 *μ*L of sterile commercial 0.9% saline, at the same conditions. Spleen cells were analyzed 14 days after HEW implantation. (c) Gates strategy used for flow cytometry analysis.

As described above, in our previous studies, we established two* P. acnes*- or PS-treatment protocols, which potentiated (Protocol 1) or suppressed (Protocol 2) the Th2 response to OVA after footpad challenge [24, 26], and the same protocols were used in the present work. In Protocol 1 (Th2 potentiation), mice were subcutaneously injected with* P. acnes* (140 *μ*g/350 *μ*L/animal) or PS (25 *μ*g/350 *μ*L/animal) once a week, during 3 weeks, and HEW was implanted concomitantly to the last* P. acnes* or PS injection (Figure 1(a)). In Protocol 2 (Th2 suppression), HEW was implanted 1 week after the third dose of* P. acnes* or PS (Figure 1(b)). For each protocol, the respective control group was treated with 350 *μ*L of sterile commercial 0.9% saline, at the same conditions.

Herein, mice were treated according to both protocols described above but not submitted to footpad challenge. Instead, at the moment in which OVA challenge should be done, that is, 14 days after HEW implantation, spleen cells were collected and activation status of APCs was analyzed by flow cytometry (Figure 1).

### 2.5. Expression of Costimulatory Molecules, TLRs, and Cytokines Synthesis by Spleen Cells

Spleen cells from control,* P. acnes*- or PS-treated mice were obtained 14 days after HEW implantation. Cellular viability and concentration (total absolute number of cells/mL) were determined by counting with Trypan Blue. Cells (1 × 10^6^/100 *μ*L/tube) were then incubated for 30 minutes at 4°C with normal mouse serum (5% in PBS) to block Fc receptors. After washing, cells were labeled for 1 hour at 4°C with fluorochrome-conjugated monoclonal anti-mouse antibodies (BD Pharmingen, CA, USA) to determine the expression of costimulatory molecules (CD40, CD80, and CD86), TLRs (TLR2, TLR4, and intra- and extracellular TLR9), and cytokines synthesis (IL-4 and IL-12) by B lymphocytes (CD19^+^ CD23^+^), macrophages (CD11b^+^ F4/80^+^), and dendritic cells (CD11c^+^ MHC II^+^). TLR9 was analyzed not only intracellularly, but also extracellularly, since the presence of this receptor on the cell membrane of human peripheral blood B lymphocytes and of mouse peritoneal B-1b cells has already been described [34, 35].

When intracellular staining had to be performed (intracellular TLR9 and cytokines), cells were washed and fixed (1% paraformaldehyde) after extracellular labeling and then permeabilized with Triton-X-100 (0.2% in PBS) for 6 minutes, followed by intracellular labeling for 40 minutes, at room temperature. Cells were then washed and analyzed in a FACSCalibur flow cytometer, using the CellQuest Pro software (Becton Dickinson, CA, USA).

Percentages of positive cells were converted to absolute number (cells/mL) based on the total cell concentration obtained by Trypan Blue counting, once both* P. acnes* and PS treatments were able to increase the absolute number of total spleen leukocytes (data not shown). The absolute numbers of B lymphocytes, macrophages, and dendritic cells positive for each molecule were added to obtain the total number of spleen APCs positive for such molecule. Expression level of costimulatory molecules, TLRs, and cytokines synthesis was also determined by mean fluorescence intensity (MFI) and individually analyzed in each population. Gates strategy used in flow cytometry analysis is shown in Figure 1(c).

Similarly to what was described above, intracellular staining was performed to determine the absolute number of total spleen cells positive for IL-5 synthesis, an important cytokine in type I hypersensitivity reaction, with an essential role in eosinophil recruitment [36, 37]. Gates strategy used for this analysis is shown in Figure 5(a).

### 2.6. Cytokines Detection on Cocultures of Bone Marrow-Derived Dendritic Cells (BMDC) and OVA-Primed T Lymphocytes Stimulated* In Vitro* with* P. acnes*, PS, and/or OVA

*BMDC Achievement*. Femur and tibia from naïve F1 BALB/c x A/J mice were extracted, and their cavities were washed with RPMI (Cultilab, SP, Brazil) to obtain bone marrow cells, which were submitted to 400 g centrifugation for 5 minutes and resuspended in 1 mL of R10 (RPMI supplemented with 10% fetal cow serum). Cellular viability and concentration were determined by counting with Trypan Blue. Based on previously described protocols to obtain BMDC [21–23], cells (2 × 10^5^/mL) were cultured for 10 days at 37°C and 5% CO_2_ in R10 supplemented with GM-CSF (20 ng/mL), replacing the medium on days 3, 6, and 8. At 10th day, GM-CSF concentration was reduced to 5 ng/mL; OVA-primed T lymphocytes and* in vitro* stimuli were then added, as described below.

*OVA-Primed T Lymphocytes Enrichment*. Naïve F1 BALB/c x A/J mice were submitted to HEW implantation, and after 14 days, spleen cells were obtained and mixed to carbonyl iron (0.1 mg/mL) to increase macrophage density. After incubation at 37°C for 30 minutes, macrophage-depleted supernatant cells were collected, washed, and submitted to Histopaque (Sigma-Aldrich, MO, USA) density gradient centrifugation (*d* = 1.077 g/mL), for 30 minutes, at 400 g. Mononuclear cells-enriched ring was collected and washed with RPMI, for 5 minutes, at 400 g. Cell suspension was then transferred twice to Petri dishes, previously coated with purified anti-mouse IgM (10 *μ*g/mL), and incubated for 1 hour at 37°C and 5% CO_2_, for B cell depletion. Supernatant cells were collected, and purity was verified by flow cytometry. A T cell-enriched population (95%) was obtained and added to BMDC cultures, as described below.

*BMDC and OVA-Primed T Lymphocytes Cocultures*. At 10th day of BMDC culture, OVA-primed T lymphocytes (5 × 10^5^/mL) were added, and different* in vitro* stimuli were performed as follows: (a) cultures stimulated or not with* P. acnes* (2.5 *μ*g/mL), PS (0.5 *μ*g/mL), or OVA (17.5 *μ*g/mL) at 10th day; (b) cultures stimulated with* P. acnes* or PS, concomitantly to OVA, at 10th day; (c) cultures stimulated with* P. acnes* or PS at 10th day and OVA at 11th day. All supernatants were collected at 12th day and submitted to ELISA for IL-4, IL-5, IL-12, IL-17, and IFN-*γ* detection.

*ELISA for Cytokines Detection*. IL-17 detection was performed according to the manufacturer's instructions (eBioscience, CA, USA). For the other cytokines, plates were coated with 50 *μ*L/well of purified monoclonal anti-mouse IL-4 (1 *μ*g/mL), anti-mouse IL-5 (2 *μ*g/mL), anti-mouse IL-12 (4 *μ*g/mL), or anti-mouse IFN-*γ* (6 *μ*g/mL) (R&D Systems, MN, USA) overnight, at room temperature, and washed three times with PBS-Tween 20 0.05% (200 *μ*L/well). After blocking free sites with PBS-BSA 1% (200 *μ*L/well), for 1 hour, at room temperature, plates were washed again, and recombinant cytokines (15 pg/mL to 4,000 pg/mL) (R&D Systems) or samples (50 *μ*L/well) were incubated for 2 hours at room temperature. After washing, biotinylated monoclonal anti-mouse IL-4 (400 ng/mL), anti-mouse IL-5 (400 ng/mL), anti-mouse IL-12 (200 ng/mL), or anti-mouse IFN-*γ* (400 ng/mL) (R&D Systems) was added (50 *μ*L/well) and incubated for 2 hours, at room temperature. Plates were washed again and incubated with 50 *μ*L/well of HRP-conjugated streptavidin (1 : 4,000) (BD Pharmingen) for 30 minutes, at room temperature. After washing, peroxidase activity was assessed with OPD-substrate and stopped with 4 N H_2_SO_4_. Optical density was determined at 492 nm. Cytokines levels were calculated based on recombinant cytokines curves.

### 2.7. Statistical Analysis

The statistical differences between control and treated groups were analyzed by one-way ANOVA followed by Tukey's multiple comparison posttest, using GraphPad Prism software (GraphPad Software, CA, USA). Differences were taken as statistically significant when *P* < 0.05.

## 3. Results

### 3.1. *P. acnes* or PS Treatments according to Protocol 1 (Th2 Potentiation) Increased the Absolute Number and Expression Level of CD40^+^, CD80^+^, and CD86^+^ Spleen APCs

Absolute numbers of spleen B lymphocytes, macrophages, and dendritic cells CD40^+^, CD80^+^, or CD86^+^, from each treated group, were added to obtain the total number of spleen APCs positive for these costimulatory molecules, while expression level (MFI) was analyzed individually for each population.

**Figure 2:**
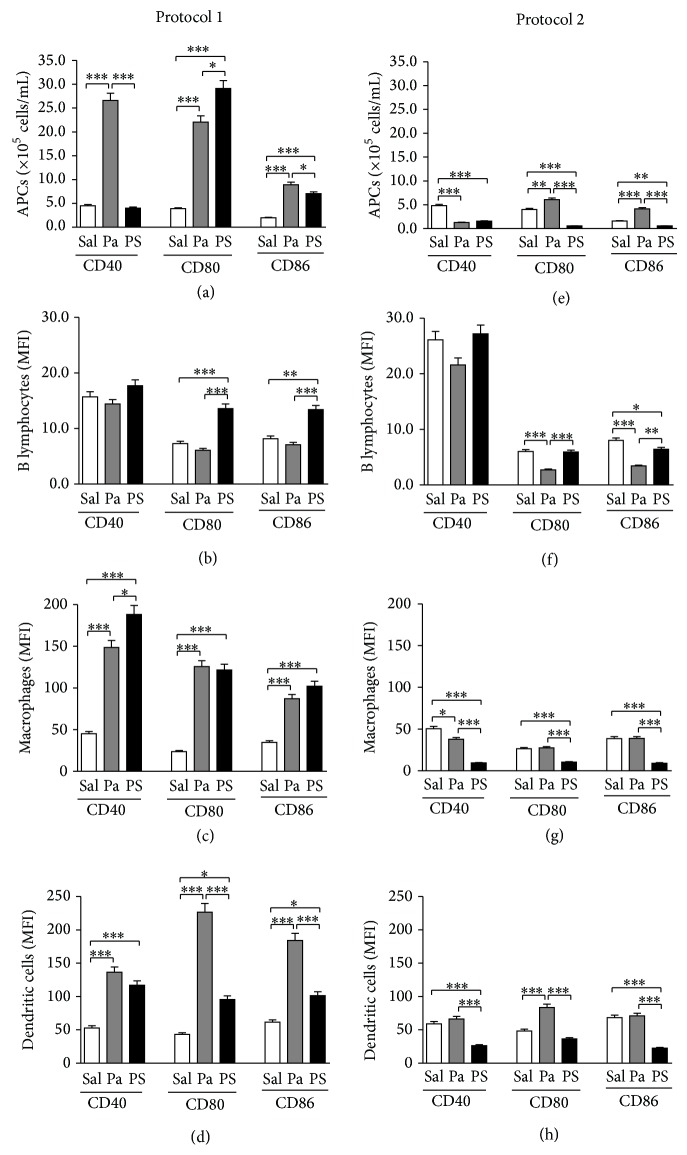
Expression of costimulatory molecules by spleen antigen-presenting cells. Spleen cells from mice treated with saline (Sal),* P. acnes* (Pa), or PS were obtained 14 days after HEW implantation and stained with fluorochrome-conjugated monoclonal anti-mouse antibodies to determine the expression of CD40, CD80, and CD86 by B lymphocytes, macrophages, and dendritic cells. Percentages obtained by flow cytometric analysis were converted to absolute numbers of B lymphocytes, macrophages, and dendritic cells positive for each molecule, which were added to obtain the total number of spleen APCs positive for such molecule in Protocols 1 (a) and 2 (e). Expression levels were also determined by mean fluorescence intensity (MFI) and individually analyzed in B lymphocytes ((b) and (f)), macrophages ((c) and (g)), and dendritic cells ((d) and (h)) for Protocols 1 and 2, respectively. ^*^ *P* < 0.05; ^**^ *P* < 0.01; ^***^ *P* < 0.0001.

We observed that* P. acnes* and PS treatments according to Protocol 1 increased the absolute number of APCs expressing costimulatory molecules, except CD40 in PS group (Figure 2(a)), and also enhanced their expression levels by macrophages and dendritic cells (Figures 2(c) and 2(d)). PS also upregulated the expression of CD80 and CD86 by B lymphocytes (Figure 2(b)).

### 3.2. In General,* P. acnes* or PS Treatments according to Protocol 2 (Th2 Suppression) Slightly Increased or Even Decreased the Absolute Number of Costimulatory Positive Spleen APCs and Reduced Their Expression Levels

*P. acnes* treatment according to Protocol 2 enhanced the number of APCs expressing CD80 and CD86, but at a much lower level than Protocol 1, besides having impaired the number of CD40^+^ cells (Figure 2(e)). PS decreased the number of cells expressing all three analyzed molecules (Figure 2(e)). In addition, there was also a decrease in the expression level of CD80 and CD86 by B lymphocytes from* P. acnes*-treated group (Figure 2(f)), as well as CD40 by macrophages (Figure 2(g)), and only a slight increase of CD80 by dendritic cells (Figure 2(h)). PS downregulated the expression of the three molecules by macrophages (Figure 2(g)), CD40 and CD86 by dendritic cells (Figure 2(h)), and CD86 by B lymphocytes (Figure 2(f)).

### 3.3. Both Protocols Increased the Number of APCs Expressing TLRs, but the Expression Levels of These Molecules Were Predominantly Enhanced in Mice Submitted to Protocol 2 (Th2 Suppression), Mainly in* P. acnes* Group

**Figure 3:**
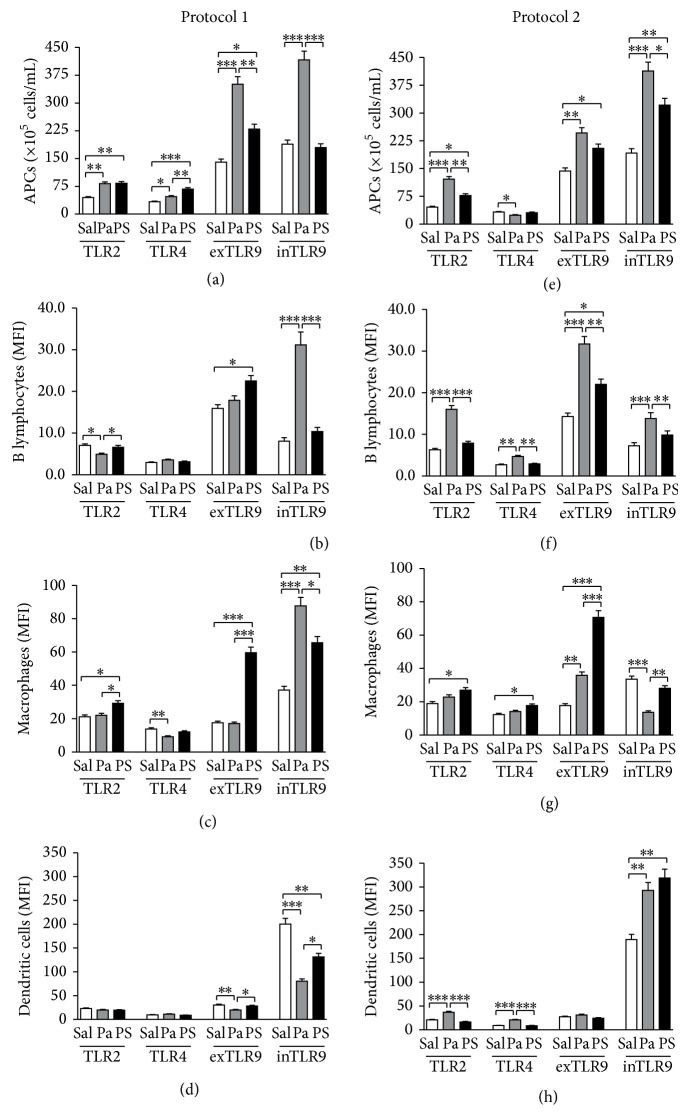
Expression of toll-like receptors by spleen antigen-presenting cells. Spleen cells from mice treated with saline (Sal),* P. acnes* (Pa), or PS were obtained 14 days after HEW implantation and stained with fluorochrome-conjugated monoclonal anti-mouse antibodies to determine the expression of TLR2, TLR4, and intra- and extracellular TLR9 by B lymphocytes, macrophages, and dendritic cells. Percentages obtained by flow cytometric analysis were converted to absolute numbers of B lymphocytes, macrophages, and dendritic cells positive for each receptor, which were added to obtain the total number of spleen APCs positive for such receptor in Protocols 1 (a) and 2 (e). Expression levels were also determined by mean fluorescence intensity (MFI) and individually analyzed in B lymphocytes ((b) and (f)), macrophages ((c) and (g)), and dendritic cells ((d) and (h)) for Protocols 1 and 2, respectively. ^*^ *P* < 0.05; ^**^ *P* < 0.01; ^***^ *P* < 0.0001.

In mice submitted to Protocol 1, there was an increase in the absolute number of spleen APCs expressing TLR2, TLR4, and extra- and intracellular TLR9 in* P. acnes* and PS groups, except intracellular TLR9 for PS group (Figure 3(a)).* P. acnes* treatment increased the expression level of intracellular TLR9 by B lymphocytes and macrophages, while PS increased extracellular TLR9 in these populations (Figures 3(b) and 3(c)). PS also upregulated the expression of TLR2 and intracellular TLR9 by macrophages (Figure 3(c)). On the other hand,* P. acnes* decreased the expression of TLR2 by B lymphocytes (Figure 3(b)), TLR4 by macrophages (Figure 3(c)), and extra- and intracellular TLR9 by dendritic cells (Figure 3(d)). PS only decreased the expression of intracellular TLR9 by dendritic cells (Figure 3(d)).

In Protocol 2, there was also an increase in the absolute number of spleen APCs expressing TLR2 and extra- and intracellular TLR9 in* P. acnes* and PS groups, but not TLR4 (Figure 3(e)). Differently from Protocol 1,* P. acnes* upregulated the expression of all TLRs by B lymphocytes (Figure 3(f)), extracellular TLR9 by macrophages (Figure 3(g)), and TLR2, TLR4, and intracellular TLR9 by dendritic cells (Figure 3(h)). PS also increased the expression of extracellular TLR9 by B lymphocytes (Figure 3(f)), TLR2, TLR4, and extracellular TLR9 by macrophages (Figure 3(g)), and intracellular TLR9 by dendritic cells (Figure 3(h)).

### 3.4. *P. acnes* and PS Treatments according to Protocol 1 (Th2 Potentiation) Increased IL-4^+^ and Decreased IL-12^+^ Spleen APCs Absolute Number

**Figure 4:**
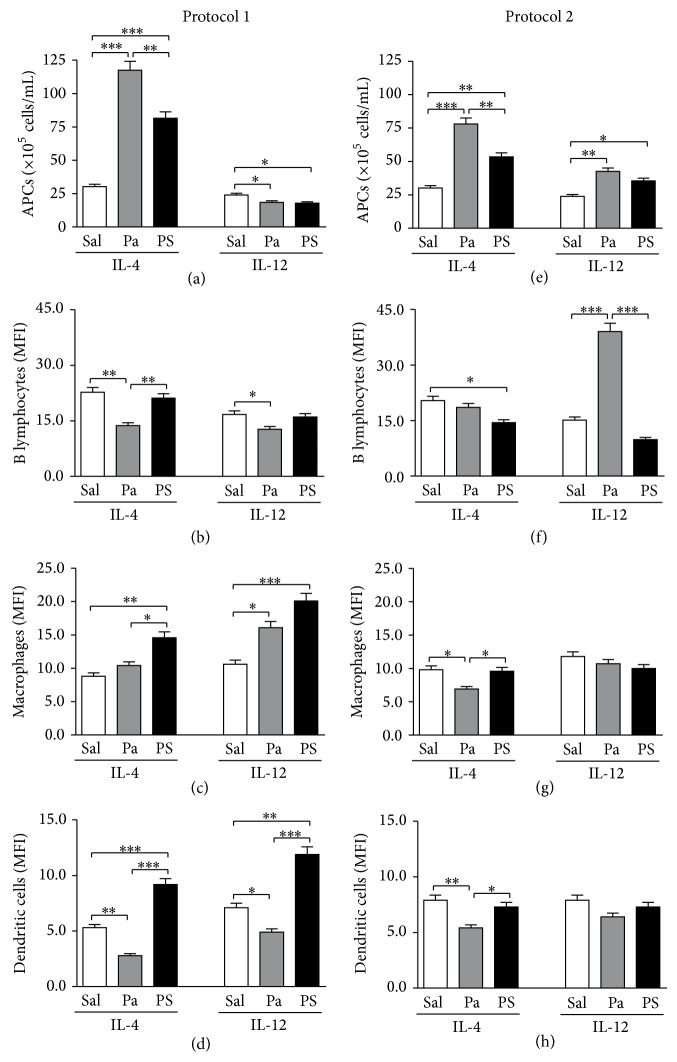
Cytokines synthesis by spleen antigen-presenting cells. Spleen cells from mice treated with saline (Sal),* P. acnes* (Pa), or PS were obtained 14 days after HEW implantation and stained with fluorochrome-conjugated monoclonal anti-mouse antibodies to determine the intracellular synthesis of IL-4 and IL-12 by B lymphocytes, macrophages, and dendritic cells. Percentages obtained by flow cytometric analysis were converted to absolute numbers of B lymphocytes, macrophages, and dendritic cells positive for each cytokine, which were added to obtain the total number of spleen APCs positive for such cytokine in Protocols 1 (a) and 2 (e). Production levels were also determined by mean fluorescence intensity (MFI) and individually analyzed in B lymphocytes ((b) and (f)), macrophages ((c) and (g)), and dendritic cells ((d) and (h)) for Protocols 1 and 2, respectively. ^*^ *P* < 0.05; ^**^ *P* < 0.01; ^***^ *P* < 0.0001.

Confirming the potentiation of the Th2 response to OVA, an increase in the number of spleen APCs synthesizing IL-4 in mice treated with* P. acnes* or PS according to Protocol 1 was observed, with a concomitant decrease in the number of IL-12^+^ cells (Figure 4(a)). Interestingly,* P. acnes* induced lower levels of these two cytokines on B lymphocytes and dendritic cells (Figures 4(b) and 4(d)) but increased the production of IL-12 by macrophages (Figure 4(c)). On the other hand, PS increased the production of IL-4 and IL-12 by macrophages and dendritic cells (Figures 4(c) and 4(d)).

### 3.5. *P. acnes* and PS Treatments according to Protocol 2 (Th2 Suppression) Increased Both IL-4^+^ and IL-12^+^ Spleen APCs Absolute Number, but IL-4^+^ Cells Increment Was Not so Pronounced as in Protocol 1 (Th2 Potentiation)

In Protocol 2, both treatments also elevated the number of APCs synthesizing IL-4, but this increase was not so marked as the one observed in Protocol 1 (Figure 4(e)). Besides, differently from Protocol 1,* P. acnes* and PS treatments according to Protocol 2 induced an increase in the number of APCs positive for IL-12 (Figure 4(e)), confirming the suppression of Th2 response.* P. acnes* treatment also increased the production of IL-12 by B lymphocytes (Figure 4(f)) and decreased IL-4 by macrophages and dendritic cells (Figures 4(g) and 4(h)), while PS decreased the synthesis of IL-4 by B cells (Figure 4(f)).

### 3.6. The Numbers of IL-5^+^ Spleen Cells Confirmed the Th2 Potentiation in Protocol 1 and the Suppression in Protocol 2 by Both* P. acnes* and PS Treatments

**Figure 5:**
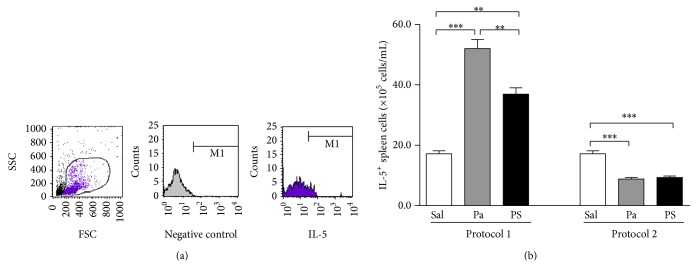
IL-5 synthesis by spleen cells. Spleen cells from mice treated with saline (Sal),* P. acnes* (Pa), or PS were obtained 14 days after HEW implantation and intracellularly stained with fluorochrome-conjugated monoclonal anti-IL-5 antibody. Percentages obtained by flow cytometric analysis (a) were converted to absolute numbers of IL-5^+^ spleen cells (b). ^**^ *P* < 0.01; ^***^ *P* < 0.0001.

*P. acnes* and PS treatments according to Protocol 1 also increased the number of total spleen cells positive for IL-5, unlike the one observed in Protocol 2, in which* P. acnes* and PS treatments decreased the number of IL-5^+^ cells (Figure 5(b)). These data also confirm Th2 potentiation response in Protocol 1 and Th2 suppression in Protocol 2.

### 3.7. Higher Levels of IL-5 and IL-17 Were Detected in Cocultures of Bone Marrow-Derived Dendritic Cells (BMDC) and OVA-Primed T Lymphocytes Submitted to Protocol 1-Like (Th2 potentiation), Compared to Lower Levels in Protocol 2-Like (Th2 Suppression)

In order to confirm the direct effect of* P. acnes* and PS on antigen-presenting cells in this model of type I hypersensitivity reaction, we performed a coculture of BMDC and OVA-primed T lymphocytes, differently stimulated with* P. acnes*, PS, and/or OVA, analyzing the pattern of cytokines released in the culture supernatant.

BMDC were obtained after culturing bone marrow cells for 10 days with GM-CSF. At 10th culture day of BMDC, OVA-primed T lymphocytes were added. To reproduce the potentiation protocol (Protocol 1-like), cocultures were stimulated with* P. acnes* and OVA or PS and OVA at 10th day, and supernatants were analyzed at 12th day. To reproduce the suppression protocol (Protocol 2-like), cocultures were stimulated with* P. acnes* or PS at 10th day and with OVA at 11th day and supernatants were analyzed at 12th day.

**Figure 6:**
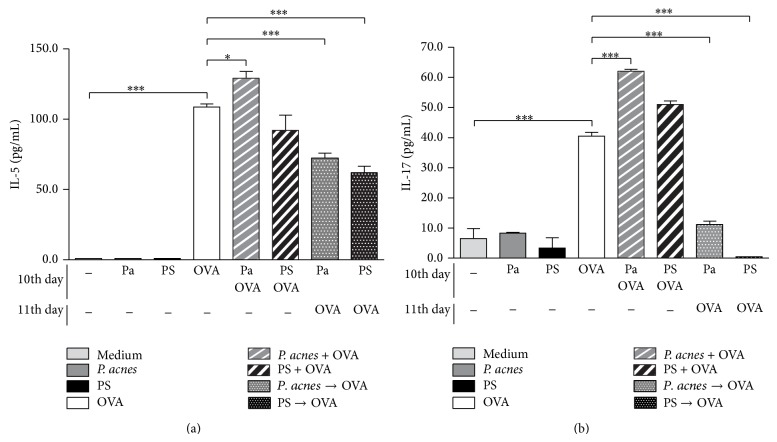
Cytokines production in cocultures of bone marrow-derived dendritic cells and OVA-primed T lymphocytes. Bone marrow cells from naïve F1 BALB/c x A/J mice were cultured during 10 days with GM-CSF, for dendritic cell differentiation. At 10th day, enriched OVA-primed T lymphocytes, obtained from the spleen of HEW-implanted F1 BALB/c x A/J mice, were added to the cultures. The following* in vitro* stimuli were performed:* P. acnes*, PS, or OVA at 10th day;* P. acnes* or PS, concomitantly to OVA, at 10th day (*P. acnes* + OVA and PS + OVA, resp.);* P. acnes* or PS at 10th day and OVA at 11th day (*P. acnes* → OVA and PS → OVA, resp.). Supernatants were collected at 12th day and submitted to ELISA for IL-5 (a) and IL-17 (b) detection. ^*^ *P* < 0.05; ^***^ *P* < 0.0001.

IL-4, IL-12, and IFN-*γ* were not detected in any culture (data not shown).* In vitro* stimulus with OVA alone (antigen-specific positive control) induced the release of detectable levels of IL-5, when compared to the negative controls (medium,* P. acnes* or PS) (Figure 6(a)). There was a significant increase in these levels when cocultures were submitted to Protocol 1-like (*P. acnes* + OVA), but a significant decrease when using the Protocol 2-like (*P. acnes* → OVA or PS → OVA) (Figure 6(a)). Similar results were observed for the production of IL-17, whose levels were elevated in cultures submitted to the potentiation protocol (*P. acnes* + OVA) and drastically impaired in the suppression protocol (*P. acnes* → OVA or PS → OVA) (Figure 6(b)). These data suggest that* P. acnes* or PS can directly act on antigen-presenting cells to modulate the Th2 response to OVA.

## 4. Discussion

In previous studies from our group, we demonstrated that, depending on the treatment protocol,* P. acnes* or PS could suppress or enhance the Th2 response to OVA in a murine model of type I hypersensitivity reaction [24, 26]. Herein, we investigated the mechanisms of action of* P. acnes* and PS in Th1/Th2 polarization, focusing on the immunophenotype and the activation status of APCs, which are responsible for driving the T helper response.

As previously described, Protocols 1 (Th2 potentiation) and 2 (Th2 suppression) differ only at the time of HEW implantation [24, 26]. Therefore, we hypothesized that* P. acnes* and PS could modulate the activation status of APCs during sensitization phase, leading to different responses to OVA challenge. In order to confirm this hypothesis, spleen cells from mice submitted to Protocols 1 (Th2 potentiation) and 2 (Th2 suppression) were analyzed at the moment in which OVA challenge should be done, that is, 14 days after HEW implantation (Figures 1(a) and 1(b)).

The activation status of spleen B lymphocytes, macrophages, and dendritic cells was analyzed by the expression of costimulatory molecules, TLRs, and cytokines production, considering both the number of positive cells and the expression level of each molecule analyzed.

According to our and others' previous publications, the better way to represent the modulatory effects of* P. acnes* and PS is analyzing the absolute number of cells, rather than the percentage, once both treatments are able to increase the total number of leukocytes in different organs, according to the inoculation route. In fact, among other organs,* P. acnes* increases the number of leukocytes in spleen, liver, and peritoneal exudate [13, 16, 28].

A great increase in the number of APCs expressing costimulatory molecules in* P. acnes* and PS groups submitted to Protocol 1 (Th2 potentiation) was observed, except CD40 in PS group (Figure 2(a)), as well as upregulation of the three molecules, mainly by macrophages and dendritic cells (Figures 2(b) to 2(d)). On the other hand, in Protocol 2 (Th2 suppression), there was little increase or even decrease in the number of positive APCs (Figure 2(e)), as well as downregulation of CD40, CD80, and CD86 (Figures 2(f) to 2(h)).  Table 1(a) summarizes the effects of* P. acnes* and PS on the expression levels of costimulatory molecules and clearly shows that both treatments mainly upregulate these molecules in Protocol 1 and downregulate in Protocol 2.

These results suggest that higher number of positive APCs and higher expression levels of costimulatory molecules could be related to potentiation of Th2 response to OVA by* P. acnes* and PS. In fact, several studies have demonstrated the role of costimulatory molecules in different models of Th2 responses. Lindell et al. (2008) verified that B cells from allergen-challenged mice upregulated the expression of CD40, CD80, and CD86 and induced the production of Th2 cytokines by T cells [38]. It has also been demonstrated that CD80 and CD86 are critical and potent stimulators of Th2 differentiation* in vitro* [39] and that these molecules are essential for the generation of Th2 cells during the sensitization phase in a murine model of asthma [40]. Similarly, it has been shown that the expression of CD40 by dendritic cells is essential for the initiation of Th2 responses [22]. Moreover, several data revised by Lenschow et al. (1996) indicate that higher stimulation of CD28 by B7 molecules is related to the induction of Th2 responses [41].

On the other hand, we hypothesize that the downregulation of costimulatory molecules observed in Protocol 2 can be related to Th2 suppression. It is important to note that the downregulation of costimulatory molecules does not mean that APCs are less activated, because other parameters, such as TLRs expression and cytokines production, have to be considered in order to determine the activation status of these cells.

Concerning the effects of PS on the modulation of CD40, it is important to note that the modulation of the number of CD40^+^ cells is not necessarily related to the modulation of the CD40 levels in cell membrane, and the same applies to all the other molecules studied. Besides, differences observed between* P. acnes* and PS treatments can be explained by the presence of other compounds in the whole bacterium, such as proteins and lipids, which could be responsible for the increase in the CD40^+^ population in Protocol 1, explaining why the same effect is not observed in PS group.

*P. acnes* and PS also modulated the number of APCs expressing TLRs, but the differences between Protocols 1 (Th2 potentiation) and 2 (Th2 suppression) were not as pronounced as that observed for costimulatory molecules, except for TLR4^+^ cells, which were increased in Protocol 1 (Figure 3(a)) and decreased in Protocol 2 (Figure 3(e)). On the other hand, as shown in Figure 3 and summarized in Table 1(b), the expression level of TLRs was enhanced in Protocol 2 (Th2 suppression) rather than Protocol 1 (Th2 potentiation), mainly in* P. acnes*-treated groups. These data suggest that higher expression of these receptors could be related to the suppression of Th2 responses. Indeed, several studies have shown the relationship between TLR signaling and the induction of Th1 responses or suppression of Th2 pattern in allergic diseases. In farmers' families, LPS exposure and higher levels of TLR2 mRNA were related to lower incidence of allergic diseases [42, 43]. TLR2 ligands were also able to inhibit Th2 responses in house dust mite allergic patients [44] and TLR4 signaling by LPS suppressed asthma-like responses in mice [45]. It was also shown that TLR9 intracellular signaling by CpG induced the release of Th1 cytokines [46] and promoted IgG2a switch, which is related to Th1 pattern [47]. Although our results do not show a clear Th1 pattern in Protocol 2, we hypothesize that the Th2 suppression could also be related to higher expression of TLRs by APCs in* P. acnes* group.

On the other hand, PS treatment did not induce distinct patterns of TLRs levels between Protocols 1 and 2, as clearly shown in Table 1(b). Therefore, we believe that the effects induced by PS in Th2 potentiation or suppression may be only related to the expression of costimulatory molecules, while the effects induced by* P. acnes* involve a more complex mechanism, including not only the expression of costimulatory molecules, but also the levels of TLRs. However, we cannot exclude other molecules and receptors which were not analyzed in the present work.

The modulation of Th1 and Th2 responses by* P. acnes* and PS was also analyzed by intracellular staining of IL-4 and IL-12 on APCs. In Protocol 1,* P. acnes* and PS treatments increased the number of IL-4^+^ APCs and decreased the number of IL-12^+^ (Figure 4(a)), confirming the potentiation of Th2 response. In Protocol 2, there was little increase of IL-4^+^ cells and also increase of IL-12^+^, indicating the suppression of Th2 response, but not changing to a Th1 pattern, since the number of IL-4^+^ cells remained higher than the number of IL-12^+^ (Figure 4(e)). The production level of these cytokines by APCs did not show a defined pattern when comparing the two protocols (Figure 4). We believe that, in our model, maybe the modulation of the intracellular levels of cytokines in APCs is not as relevant as the number of producing cells. The most abundant APC population in spleen is composed of B lymphocytes, which highly impacted the total number of APCs (data not shown). Interestingly, MFI of IL-12^+^ B lymphocytes is decreased in* P. acnes* treatment according to Protocol 1 (Figure 4(b)) and increased in Protocol 2 (Figure 4(f)). The same is not observed in PS groups, probably because the whole bacterium has other components that can be responsible for this specific effect.

Although the production of IL-4 and IL-12 by spleen APCs does not show a clear Th1 pattern in Protocol 2, we have already demonstrated the induction of intracellular and serum IFN-*γ* in mice treated with* P. acnes* according to this protocol [24]. Another important effect of* P. acnes* is its capacity to induce the production of IL-18 [11]. Therefore, maybe the combination of IL-12 and IL-18 production by APCs could be responsible for directing the T helper response, resulting in the release of IFN-*γ*. The production of IL-18 in this model needs further investigation.

Another essential cytokine in this model is undoubtedly IL-5, whose production was analyzed in the total spleen population, certainly including IL-5-producing T lymphocytes, which are the main IL-5 producer cells. The analysis of IL-5^+^ spleen cells strongly correlated to previous data, since* P. acnes* and PS increased the number of IL-5^+^ cells in Protocol 1 (Th2 potentiation) and decreased in Protocol 2 (Th2 suppression) (Figure 5). IL-5 induces maturation, survival, and chemoattraction of eosinophils and enhances their effector functions, playing a pivotal role in allergic inflammation [36, 37]. In fact, in our previous data,* P. acnes* and PS treatments had a great impact on eosinophilia in the type I hypersensitivity model, inducing enhanced eosinophil infiltration in Protocol 1 and inhibiting it in Protocol 2 [24, 26].

Then, even with little differences in the production of IL-4 and IL-12 by APCs, both protocols induced significant differences on IL-5 production and eosinophilia. We believe IL-5 is an essential cytokine involved with the biological effects observed in this model, while IL-4 and IL-12 production by APCs might not be an essential factor for potentiation or suppression of the Th2 response. Therefore, in this model, the differential expression of costimulatory molecules and TLRs by B lymphocytes, macrophages, and dendritic cells seems to be more relevant for Th1/Th2 direction by* P. acnes* and PS than the production of cytokines by these cells.

Our results suggest that APCs can be directly modulated by* P. acnes* and PS in this model, which is in agreement with other studies showing direct activation of innate immune cells by* P. acnes* via TLR2 and TLR9 [15, 16]. In order to confirm this hypothesis of direct activation of APCs, we differently stimulated BMDC with* P. acnes*, PS, and/or OVA and cocultured these cells with OVA-primed T lymphocytes, analyzing the pattern of cytokines released in the culture supernatants. Interestingly, only IL-5 and IL-17 were detected in these cultures, although we have shown the production of IL-4 and IL-12 by APCs in Figure 4. It is clear for us that these cocultures did not represent exactly the same conditions obtained* in vivo*, concerning the microenvironment and treatment periods, for example, which can explain the absence of IL-4 and IL-12* in vitro*. Besides, it is not reasonable to compare the levels of intracellular cytokines, which may not necessarily be released, with the levels of cytokines in culture supernatants. Therefore, a better analysis of* in vitro* results should include the intracellular cytokines staining, which needs further investigation.

Both IL-5 and IL-17 were increased in the* in vitro* potentiation protocol (Protocol 1-like) and decreased in the* in vitro* suppression protocol (Protocol 2-like) (Figure 6). As mentioned above, IL-5 is involved with eosinophilia and plays an essential role in allergic inflammation [36, 37]. On the other hand, IL-17 has been implicated in noncanonical forms of asthma, where neutrophilia is more evident than eosinophilia [48]. Interestingly, in our previous studies using this hypersensitivity model, although eosinophilia in control (saline) groups is considered intense, these cells are not the only population present in the cellular infiltrate [24, 26], and the remaining cells are mainly neutrophils (data not shown). This may be an indication of the participation of IL-17 in this model. In fact, clinical observations have suggested the coexistence of both Th2 and Th17 phenotypes in the same patient [48], and a new population of CD4^+^ T lymphocytes which produces both IL-4 and IL-17 has been recently identified [49]. Therefore, it is possible that our model could induce both patterns, and future functional studies will certainly contribute to better understand the roles of IL-5 and IL-17 in this model.

## 5. Conclusions

Our data indicate that* P. acnes* can directly act on antigen-presenting cells, mainly modulating the expression of costimulatory molecules and TLRs, and that PS is an important bacterium compound involved in these immunomodulatory effects.

In our model, this differential expression of costimulatory molecules and TLRs by APCs seems to be essential, rather than the production of IL-4 and IL-12, for driving the T cell response. The modulation of APCs by* P. acnes* and PS results in different levels of cytokines produced by T helper cells, probably including IL-5 and IL-17, which seem to be major cytokines in this hypersensitivity model. Ultimately, different patterns of T helper responses are, then, induced, resulting in all the biological effects observed in our previous works using this model.

These results provide new and important contribution to understanding the mechanisms by which* P. acnes* and PS modulate the immune response to OVA in a type I hypersensitivity murine model.

## Figures and Tables

**(a):** 

*P*. *acnes*	Protocol 1			Protocol 2		
	B	M	DC	B	M	DC
CD40	—	●	●	—	○	—
CD80	—	●	●	○	—	●
CD86	—	●	●	○	—	—

PS	Protocol 1			Protocol 2		
	B	M	DC	B	M	DC

CD40	—	●	●	—	○	○
CD80	●	●	●	—	○	—
CD86	●	●	●	○	○	○

**(b):** 

*P*. *acnes*	Protocol 1			Protocol 2		
	B	M	DC	B	M	DC
TLR2	○	—	—	●	—	●
TLR4	—	○	—	●	—	●
exTLR9	—	—	○	●	●	—
inTLR9	●	●	○	●	○	●

PS	Protocol 1			Protocol 2		
	B	M	DC	B	M	DC

TLR2	—	●	—	—	●	—
TLR4	—	—	—	—	●	—
exTLR9	●	●	—	●	●	—
inTLR9	—	●	○	—	—	●
